# Camera‐trap data do not indicate scaling of diel activity and cathemerality with body mass in an East African mammal assemblage

**DOI:** 10.1002/ece3.8090

**Published:** 2021-09-09

**Authors:** Marcus Clauss, Miriam Scriba, John Kioko, Jörg U. Ganzhorn, Christian Kiffner

**Affiliations:** ^1^ Clinic for Zoo Animals, Exotic Pets and Wildlife Vetsuisse Faculty University of Zurich Zurich Switzerland; ^2^ Animal Ecology and Conservation Institute of Zoology Universität Hamburg Hamburg Germany; ^3^ Center For Wildlife Management Studies The School For Field Studies Karatu Tanzania; ^4^ Junior Research Group Human‐Wildlife Conflict & Coexistence Leibniz Centre for Agricultural Landscape Research (ZALF) Müncheberg Germany

**Keywords:** body mass, camera trap, cathemerality, energy budget, temporal niche

## Abstract

Diel activity patterns of animal species reflect constraints imposed by morphological, physiological, and behavioral trade‐offs, but these trade‐offs are rarely quantified for multispecies assemblages. Based on a systematic year‐long camera‐trap study in the species‐rich mammal assemblage of Lake Manyara National Park (Tanzania), we estimated activity levels (hours active per day) and circadian rhythms of 17 herbivore and 11 faunivore species to determine the effects of body mass and trophic level on activity levels and cathemerality (the degree to which species are active throughout the day and night). Using generalized least squares and phylogenetic generalized least squares analyses, we found no support for the hypothesis that trophic level is positively associated with activity levels. We found no support for activity levels to scale positively with body mass in herbivores or to differ between ruminants and nonruminants; in faunivores, we also did not detect relationships between body mass and activity levels. Cathemerality was positively associated with activity levels but did not scale significantly with body mass. Overall, our findings caution against trophic level or body mass‐associated generalized conclusions with regard to diel activity patterns.

## Graphical abstract

Based on camera‐trap data, we estimated diel activity levels and activity patterns of 28 mammal species in Lake Manyara National Park. Using generalized least squares and phylogenetic generalized least squares analyses, we found no support for the hypothesis (1) that trophic level is positively associated with activity levels, (2) that activity levels scale with body mass, and (3) that activity levels differ between ruminants and nonruminants; but cathemerality was positively associated with activity levels.

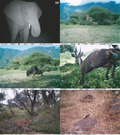

## 1 INTRODUCTION

Diel activity patterns, that is, the times when animals are active over the course of the day (e.g., circadian rhythms) and how much they are active over the course of the day (activity level), are fundamental aspects of animal behavior (Daan & Aschoff, 2001; Halberg, 1960). How animals distribute their diel activity and the duration of time during which they are active during the day largely reflect their interactions with food resources, potential mates, predators, and competitors. In their evolutionary histories, mammalian faunivores have generally shifted toward nocturnality and mammalian herbivores toward diurnality (Wu et al., 2018).

Diel activity patterns of animals can be classified into diurnal, nocturnal, crepuscular, or cathemeral (Bennie et al., 2014), and the quantitative information on which these categories are based can be gained from camera‐trap data (Rowcliffe et al., 2014). Although the relevance of this classification for very small mammals with an ultradian (and hence necessarily cathemeral) activity cycle is questionable, higher levels of diel activity are generally associated with cathemerality in larger mammals (Ramesh et al., 2015; van Schaik & Griffiths, 1996), even though quantitative assessments are still lacking.

In mammalian herbivores, body mass was positively associated with the time spent active (Belovsky & Slade, 1986) or the time spent foraging (Owen‐Smith, 1988). This also corresponds to the negative scaling of sleep time with herbivore body mass (Siegel, 2005). However, as instantaneous intake rates of mammalian herbivores scale either with metabolic body mass (Shipley et al., 1994) or linearly with body mass (Steuer et al., 2015), there is no intrinsic constraint that would force larger herbivores to spend more time foraging. If food was ubiquitous, available at more than bite depth, and of a consistent quality, larger animals would need to forage either as long as or even somewhat less than smaller herbivores, because of the similar or even slightly higher instantaneous intake capacity.

For ruminating herbivores, additional considerations of intrinsic factors apply. These animals separate the masticatory processing of their diet into the phase of ingestion that will more likely register as “activity” in motion‐triggered measures (Rowcliffe et al., 2014), and the phase of rumination that is often associated with resting. If operating at the same metabolic level, ruminating animals should therefore have lower activity levels than similar‐sized nonruminant herbivores on the same diet. For ruminants, conflicting results on the scaling of diel activity with body mass have been published, with positive relationships for foraging time (Owen‐Smith, 1988, 1992) or active time (du Toit & Yetman, 2005), negative relationships for active time (Bunnell & Gillingham, 1985; Mysterud, 1998; Pérez‐Barbería & Gordon, 1999) or feeding time (analysis of data from Belovsky & Slade, 1986; du Toit & Yetman, 2005), and no effect of body mass on time spent ruminating (Belovsky & Slade, 1986; Lauper et al., 2013; du Toit & Yetman, 2005). The inconsistency of nomenclature and methods needs to be mentioned, where terms such as “general activity,” “foraging,” or “feeding” do not necessarily represent the same behavioral categories (du Toit & Yetman, 2005).

In the last decades, systematic camera‐trap sampling evolved as a key survey method to assess wildlife populations (Beaudrot et al., 2016; O’Connell et al., 2011; Rovero & Zimmermann, 2016). Conveniently, the resulting time‐stamped pictures can be used to estimate circadian rhythms and activity levels of the photographed species (Caravaggi et al., 2017; Edwards et al., 2021; Gaynor et al., 2018; Rowcliffe et al., 2014), which often represent near complete large mammal communities (Steinbeiser et al., 2019). The conflicting findings on the relationships between diel activity characteristics and body mass are also reflected in camera‐trap studies. One camera‐trap study reported a general increase of diel activity with body mass in mammal species regardless of trophic niche (Ramesh et al., 2015). In contrast, analyses of extensive camera‐trap data across tropical forests did not find statistical support for diel activity levels to increase with body mass in mammalian herbivores (Cid et al., 2020). For faunivores, analyses of individual daily distance traveled (Carbone et al., 2005) and of camera‐trap data (Cid et al., 2020) found a positive association between diel activity levels and body mass.

**FIGURE 1:**
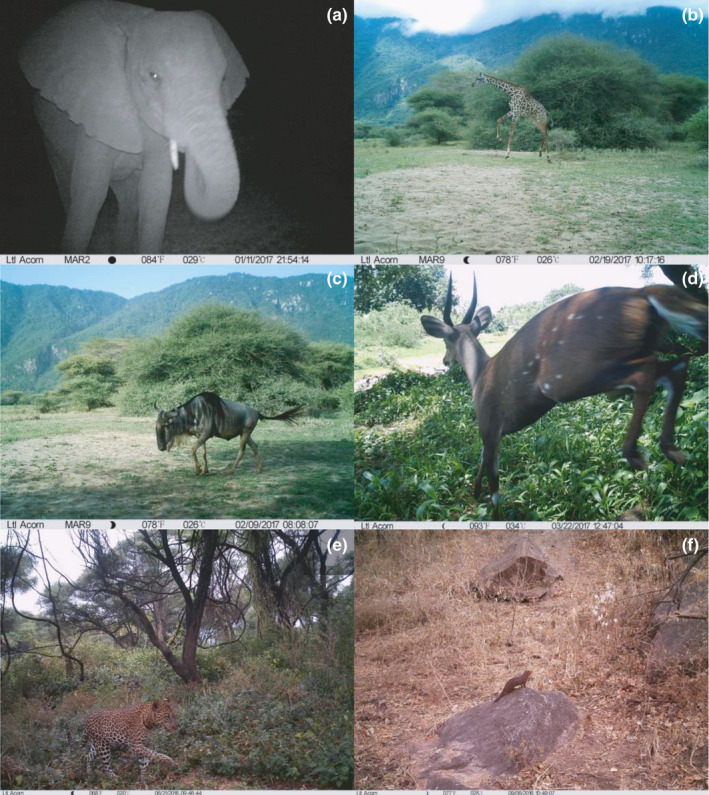
Examples of time‐stamped camera‐trap pictures of differently sized herbivores and faunivores in Lake Manyara National Park, Tanzania. (a) African elephant (*Loxodonta africana*), (b) Masai giraffe (*Giraffa camelopardalis*), (c) wildebeest (*Connochaetes taurinus*), (d) bushbuck (*Tragelaphus scriptus*), (e) leopard (*Panthera pardus*), and (f) dwarf mongoose (*Helogale parvula*)

To assess whether these associations between diel activity and body mass are manifested in a large mammal community, we conducted a systematic year‐long camera‐trap survey in Lake Manyara National Park, Tanzania. We describe species‐specific circadian rhythms and activity budgets of the species in this community (Figure 1), classify their diel activity patterns, and consider the following questions for our analyses:

1. Do activity levels increase with body mass in herbivores or faunivores?
2. Do ruminants show lower activity levels than nonruminants (because rumination, often associated with rest, is part of their digestive strategy)?
3. Are activity levels positively correlated with cathemerality? Does cathermerality therefore also increase with body mass?
4. Are there therefore negative associations of diurnality or nocturnality and body mass in herbivores and faunivores, respectively?

## 2 METHODS

### 2.1 Study area

**FIGURE 2:**
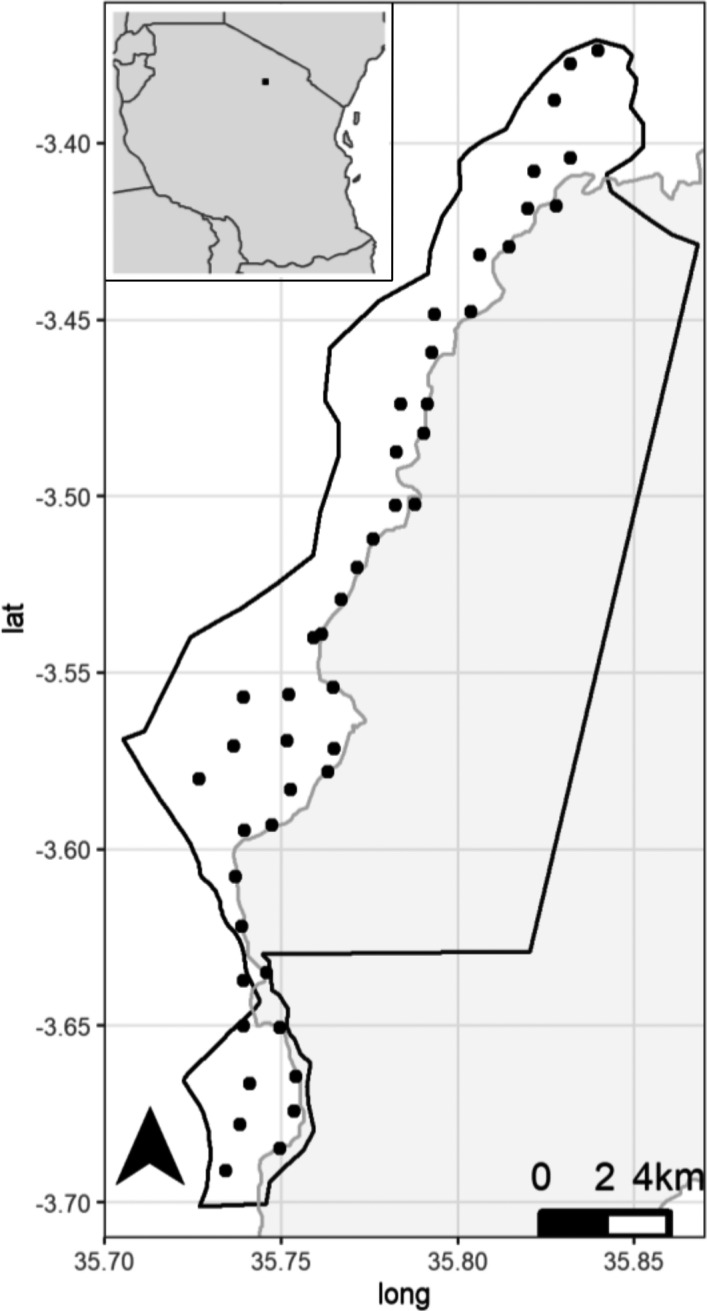
Outline of Lake Manyara (gray‐shaded area), the lowland areas of Lake Manyara National Park (LMNP), and spatial distribution of the camera traps (black dots). The inset in the top left indicates the location of LMNP within Tanzania

We conducted this study in Lake Manyara National Park (hereafter LMNP) in northern Tanzania from 6 June 2016 to 11 June 2017. LMNP is a relatively small (total land area: 428 km²) and diverse protected area along the Great Rift Escarpment. We restricted our sampling to the lowland areas (c. 168 km²), located in between Lake Manyara and the escarpment (Figure 2). The vegetation is diverse and includes alkaline grasslands near the shore of the lake, and Acacia and escarpment woodlands. Multiple seasonal rivers and a high groundwater table support lush riverine vegetation and ground water forests in some areas (Greenway & Vesey‐Fitzgerald, 1969; Loth & Prins, 1986). The park once harbored one of the highest terrestrial mammal biomass densities in the world (Prins & Douglas‐Hamilton, 1990), and, despite local extinctions (Newmark, 1996) and reductions in some megaherbivore populations (Kiffner et al., 2017), still holds a relatively species‐rich and abundant mammal community (Steinbeiser et al., 2019). Wildlife populations in LMNP are considered to be resident throughout the year (Lee & Bolger, 2017; Morrison & Bolger, 2012).

The climate is characterized as semi‐arid with a bimodal rainfall pattern. From 1958 to 2018, annual precipitation averaged 608 mm (range: 108–1203 mm; data from weather station at the LMNP headquarters). Typically, long rains occur from March to May and short rains from October to December (Prins & Loth, 1988). However, during our survey from June 2016 to June 2017, the area experienced below average amounts of precipitation during the period that is otherwise characterized as short rains (92 mm in 2016/2017 vs. 242 mm during an average short rain period).

### 2.2 Camera‐trap survey

We used remote camera traps (LTL Acorn 5210A, Zhuhai Ltl Acorn Electronics Co Ltd., Guangdong, China) to assess the activity of mammal species remotely and with minimal disturbance over the course of 1 year. The cameras record animal movement with the aid of three passive infrared motion sensors and illuminate the scene with an infrared flash during low‐light conditions. To systematically cover the study area, we superimposed a 1.5 × 1.5 km grid and deployed the cameras in or near the center of these grid cells (Figure 2). Some predefined cells were inaccessible, and we could sample a total of 46 camera stations. Because we did not have a sufficient number of camera traps to operate all stations simultaneously, we rotated the 23 available camera traps between neighboring locations approximately every 2 months. At each location, we attached one camera trap to the trunk of a suitable tree at a height of c. 0.5 m. In two locations, we attached cameras at greater tree height to avoid repeated camera damage by spotted hyenas. We set cameras to normal sensitivity, one picture per trigger, and a 1 min delay after each picture. We replaced *SD* cards and batteries on a monthly basis and did not use baits at camera stations. Pictures were catalogued in the “*Camerabase*” extension of Microsoft Access (Tobler, 2015) and identified by trained wildlife management students with the help of a field guide (Foley et al., 2014). Dates and times of pictures were checked for plausibility, and in few cases, date and time settings of the camera traps were set incorrectly. In those cases, we adjusted the time and date of the pictures from this camera trap by comparing the time stamp of the test picture (which was taken during the camera set up or maintenance) with the recorded time of the maintenance protocol.

### 2.3 Data analysis

We classified species broadly as herbivores or faunivores (Table 1). In line with similar camera‐trap research on animal activity patterns in similar systems (Havmøller et al., 2020), we removed pictures of the same species that were captured within 30 min of the first picture to increase independence of sampling events. Since camera traps record animal movement, we defined each independent record as activity, assuming that the trap rate at a given time of day is proportional to the activity level of the population at that time (Rowcliffe et al., 2014).

**TABLE 1:** Overview of sampled mammal species captured by camera traps in Lake Manyara National Park, Tanzania

Common name	Scientific name	Digestion system	Body mass (kg)	Camera‐trap events	Activity level (hours day^−1^), incl. 95% CI	Diurnal (%)	Nocturnal (%)	Activity classification	
								Bennie et al. (2014)	This study
**Herbivores**									
African elephant	*Loxodonta africana*	H	4,000	1,080	14.85 (13.06; 16.34)	52.9%	47.1%	Cathemeral	Cathemeral
Hippopotamus	*Hippopotamus amphibius*	NR	1,900	617	8.25 (6.98; 9.33)	4.4%	95.6%	Nocturnal	Nocturnal
Masai giraffe	*Giraffa camelopardalis tippelskirchi*	R	1,340	504	8.99 (7.74; 9.55)	90.7%	9.3%	Crepuscular	Diurnal
African buffalo	*Syncerus caffer*	R	550	778	10.52 (9.16; 11.97)	65.2%	34.8%	Nocturnal	Cathemeral
Zebra	*Equus quagga*	H	241.8	714	13.85 (11.73; 15.13)	67.6%	32.4%	Cathemeral	Cathemeral
Wildebeest	*Connochaetes taurinus*	R	226.5	937	8.99 (8.09; 9.74)	87.1%	12.9%	Nocturnal	Diurnal
Waterbuck	*Kobus ellipsiprymnus*	R	215	243	12.45 (10.12; 13.35)	84.8%	15.2%	Diurnal	Diurnal
Warthog	*Phacochoerus africanus*	H	82.5	579	9.31 (7.93; 10.18)	94.3%	5.7%	Diurnal	Diurnal
Impala	*Aepyceros melampus*	R	56.3	2,006	13.22 (11.98; 14.51)	72.6%	27.4%	Diurnal	Cathemeral (Diurnal)
Bushbuck	*Tragelaphus scriptus*	R	48.5	627	15.64 (13.13; 17.02)	32.2%	67.8%	Nocturnal	Cathemeral
Olive baboon	*Papio anubis*	H	28.3	2,197	8.98 (8.27; 9.41)	99.4%	0.6%	Diurnal	Diurnal
Crested porcupine	*Hystrix cristata*	H	19.5	178	9.09 (7.32; 9.60)	1.1%	98.9%	Nocturnal	Nocturnal
Red duiker	*Cephalophus natalensis*	R	13	60	5.49 (3.84; 7.69)	93.3%	6.7%	Diurnal	Diurnal
Manyara monkey	*Cercopithecus mitis manyaraensis*	H	8.6	52	7.41 (5.01; 9.65)	98.1%	1.9%	Diurnal	Diurnal
Kirk's dik‐dik	*Madoqua kirkii*	R	5.5	377	14.71 (11.97; 17.21)	54.4%	45.6%	Cathemeral	Cathemeral
Vervet monkey	*Chlorocebus pygerythrus*	H	5.1	1,232	10.19 (9.03; 10.80)	98.0%	2.0%	Diurnal	Diurnal
Bush hyrax	*Heterohyrax brucei*	NR	1.9	184	3.54 (2.98;4.22)	90.2%	9.8%	Diurnal	Diurnal
**Faunivores**									
African lion	*Panthera leo*		178.5	47	9.11 (5.75; 10.91)	8.5%	91.5%	Nocturnal	Nocturnal
Spotted hyena	*Crocuta*		65	355	9.30 (7.76; 10.32)	4.8%	95.2%	Nocturnal	Nocturnal
Leopard	*Panthera pardus*		53.3	49	12.68 (7.68; 15.56)	26.5%	73.5%	Nocturnal	Cathemeral (Nocturnal)
Black‐backed jackal	*Canis mesomelas*		10	124	12.29 (8.71; 16.20)	46.0%	54.0%	Nocturnal	Cathemeral
Honey badger	*Mellivora capensis*		9.9	39	9.39 (5.56; 13.41)	17.9%	82.1%	Nocturnal	Nocturnal
White‐tailed mongoose	*Ichneumia albicauda*		3.9	114	9.59 (6.98; 10.21)	0.0%	100.0%	Nocturnal	Nocturnal
Large‐spotted genet	*Genetta tigrina*		2.4	61	7.41 (5.10; 9.40)	1.6%	98.4%	Nocturnal	Nocturnal
Common genet	*Genetta*		1.8	142	11.09 (8.31; 11.65)	2.1%	97.9%	Nocturnal	Nocturnal
Bushy‐tailed mongoose	*Bdeogale crassicauda*		1.7	155	9.30 (7.43; 9.85)	0.0%	100.0%	Nocturnal	Nocturnal
Banded mongoose	*Mungos mungo*		0.8	303	8.13 (6.68; 9.13)	98.3%	1.7%	Diurnal	Diurnal
Dwarf mongoose	*Helogale parvula*		0.3	43	6.93 (4.35; 9.58)	97.7%	2.3%	Diurnal	Diurnal

Note

For herbivores, we indicated the digestive system as either ruminant (R) or nonruminant (nonruminant foregut fermenter NR or hindgut fermenter H). For all species, we indicated body mass, the number of independent camera‐trap events, the estimated activity levels (incl. associated 95% confidence intervals), the percentage of diurnal and nocturnal camera‐trap events as well as the activity classification based on Bennie et al. (2014) and based on this study. We classified species as cathemeral if ≥20% (or ≥30%, only indicated if deviating) of activity occurred during the nonpeak period of the 24‐hr cycle.

We conducted all analyses in R 3.6 (R Core Team, 2016). To estimate activity levels, we generated kernel density estimates of species' diel activity patterns using the *fitact* function in the *activity* package (Rowcliffe, 2019); we permutated the *fitact* function 1,000 times and estimated mean activity levels (proportion active day^−1^) and associated 95% confidence intervals. This function relies on the key assumption that all individuals in a population are active at the peak of the circadian rhythm and estimates the activity levels as a proportion of activity over the 24‐hr cycle; this method is thus independent of differences in species‐specific densities or absolute capture rates (Rowcliffe et al., 2014). We multiplied the proportional activity levels with 24 to estimate the absolute time that a species is active during a 24‐hr cycle. To visually describe circadian rhythms, we plotted the kernel density to radian time‐of‐day relationships using the *overlap* package (Meredith & Ridout, 2020); to illustrate day and night time in the activity plots, we used the average time of sunrise (06:33) and sunset (18:39) over the study period.

Species‐specific activities were separated by day and night, using sunrise and sunset as cutoff limits between the two time periods. For the 15th day of each month, we acquired the time of sunrise (range from 06:14–06:44) and sunset (18:27–18:57) at Mto wa Mbu (a town directly north of the national park) from https://www.timeanddate.com/sun/@152743 and assigned these times as cutoff limits between day and night for each month. As a continuous index of cathemerality, we used the proportion of activity for the less used phase of the day (irrespective of whether day or night). A proportion of 0 thus denotes an either exclusively diurnal or exclusively nocturnal activity, whereas a proportion of 0.5 describes a perfectly balanced cathemeral activity. To compare our data with Bennie et al. (2014), we classified diel activity patterns as diurnal, nocturnal, crepuscular, or cathemeral using the peak(s) of activity as main criteria. Because Bennie et al. (2014) only provided a qualitative definition for cathemerality (“significant activity both during daylight and night”), we classified species as cathemeral if they showed either ≥20% or ≥30% of activity during the nonpeak time of the 24‐hr cycle.

To test for scaling between activity level and body mass across trophic groups (herbivores and faunivores), we used generalized least squares (GLS) and phylogenetic generalized least squares (PGLS, with the phylogenetic signal lambda estimated by maximum likelihood) analyses to estimate the scaling of activity levels, nocturnality (proportion of activity between sunset and sunrise relative to entire activity during a 24‐hr cycle), and the index of cathemerality with body mass according to y = *a* BM*^b^* using log‐transformed data, and for assessing relationships between the cathemerality index and activity levels. Nocturnality was used rather than diurnality because no species had zero activity at night (whereas some had zero activity during the day), which was more conducive for log‐transformation. We performed analyses GLS and PGLS analyses using the packages *nlme* (Pinheiro et al., 2021) and *caper* (Orme, 2018), linking the data to a mammalian supertree (Fritz et al., 2009). For models including trophic level of digestive physiology, we also tested for a body mass x trophic niche interaction; the interaction term was never significant. To assess the effect of including the three primate species in the herbivore models, we conducted all analyses with and without the three primate species.

For comparison, we plotted our diel activity level data against that from Cid et al. (2020), after extracting values from their Figure S3.1 using the Webplot digitizer.

## 3 RESULTS

Over the course of 6,479 camera‐trap nights, we obtained a total of 13,979 independent detections of 17 herbivore species and 11 faunivore species (Table 1). The average number of independent sampling events per species was 727 (range 52–2197) for herbivores and 130 (39–355) for faunivores (Table 1). Among the herbivores, olive baboon (*Papio anubis*), impala (*Aepyceros melampus*), vervet monkey (*Chlorocebus pygerythrus*), elephant (*Loxodonta africana*), and wildebeest (*Connochaetes taurinus*) were captured most frequently. Among the faunivore species, spotted hyenas (*Crocuta crocuta*), banded mongoose (*Mungos mungo*), bushy‐tailed mongoose (*Bdeogale crassicaudata*), common genet (*Genetta tigrina*), and black‐backed jackals (*Canis mesomelas*) had the greatest number of camera‐trap events. Overall, herbivores had greater numbers of camera‐trap events than faunivores, at a ratio of 8.6:1 (Table 1).

### 3.1 Do activity levels increase with body mass in herbivores or faunivores? Do ruminants show lower activity levels than nonruminants?

**FIGURE 3:**
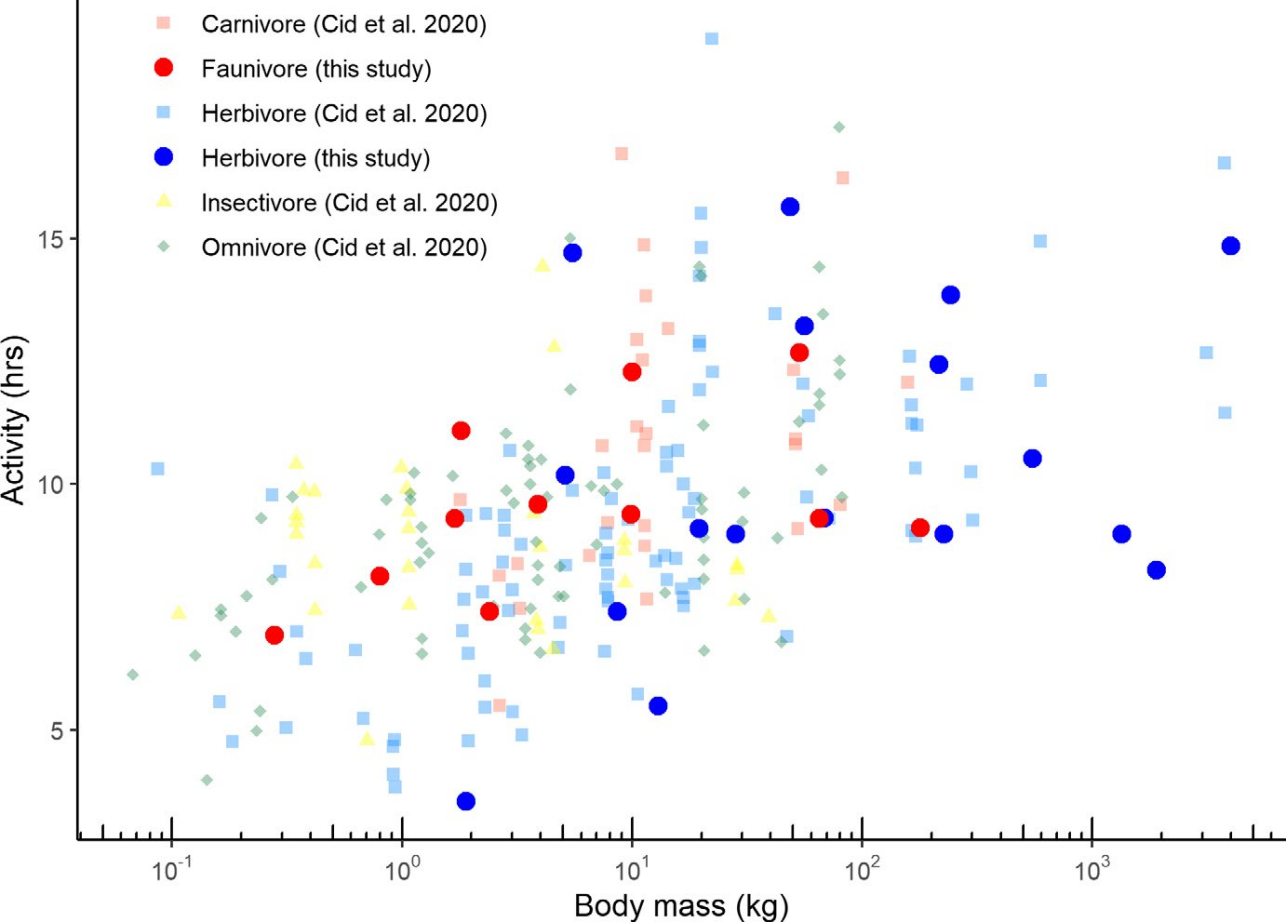
Relationship of activity levels and body mass from this study as compared to data obtained from 249 populations of terrestrial mammals in the tropics (Cid et al., 2020), distinguished as carnivores, herbivores, insectivores, and omnivores. Note the general overlap of data, that a scaling in herbivores may depend critically on including species smaller than available in the present study, and that the scaling in carnivores may depend critically on whether invertebrate and vertebrate prey are considered different trophic niches or not. Statistics for the data of this study are in Table 2

Mean diel activity levels of herbivores ranged from 3.54 hr (bush hyrax) to 15.64 hr (bushbuck *Tragelaphus scriptus*); the average of active hours in herbivores was 10.32 hr. Activity levels of faunivores ranged from 6.93 hr (dwarf mongoose *Helogale parvula*) to 12.68 hr (leopard *Panthera pardus*); the average activity level in faunivores was 9.56 hr (Table 1). Generally, our data covered a similar body mass range as that of species in Cid et al. (2020) (Figure 3).

The number of hours active scaled to BM^0.05[95% CI: 0.00,0.10]^ for all species, with no significant effect of trophic level (Table 2; Figure 3). For the herbivore species alone, there was no significant scaling (BM^0.07[−0.01,0.15]^) and no effect of being a ruminant or not. For the faunivore species alone, the scaling was also not significant (BM^0.05[−0.01,0.10]^). Accounting for the phylogenetic structure of the data did not change these findings (Table 2). Excluding the three primate species did not change the overall scaling in the complete dataset, but the exponent changed from being significant (*p* = .042 including the primates) to nonsignificant (*p* = .057 excluding the primates).

**TABLE 2:** Scaling of diel hours active with body mass (BM) according to *y* = *a* BM*^b^* or *y* = *a* BM*^b^ c*, determined by linear regression on log‐transformed data

Dataset	lambda	*a*	*p*	*b*	*p*	*c*	*p*
All	0^a^	8.1 (6.7; 9.8)	<0.001	0.05 (0.00; 0.10)	0.042	–	–
	0^a^	7.4 (5.7; 9.7)	<0.001	0.06 (0.01; 0.12)	0.030	1.1 (0.9; 1.5)	0.364
Herbivores	0^a^	7.2 (4.9; 10.5)	<0.001	0.07 (−0.01; 0.15)	0.101	–	–
	0^a^	6.8 (4.6; 10.2)	<0.001	0.07 (−0.01; 0.15)	0.129	1.2 (0.8; 1.7)	0.398
Faunivores	0^a^	8.6 (7.5; 9.9)	<0.001	0.05 (−0.01; 0.10)	0.115	–	–

Note

For the whole dataset, *c* is the factor for trophic level (multiples of faunivore as compared to herbivore); for the herbivore dataset, *c* is the factor for digestive physiology (multiples of ruminant as compared to nonruminant). Parameters are given with their 95% confidence intervals in parentheses.

^a^ PGLS yielded the same result as GLS because lambda was estimated by maximum likelihood as zero

### 3.2 Are activity levels positively correlated with cathemerality?

**FIGURE 4:**
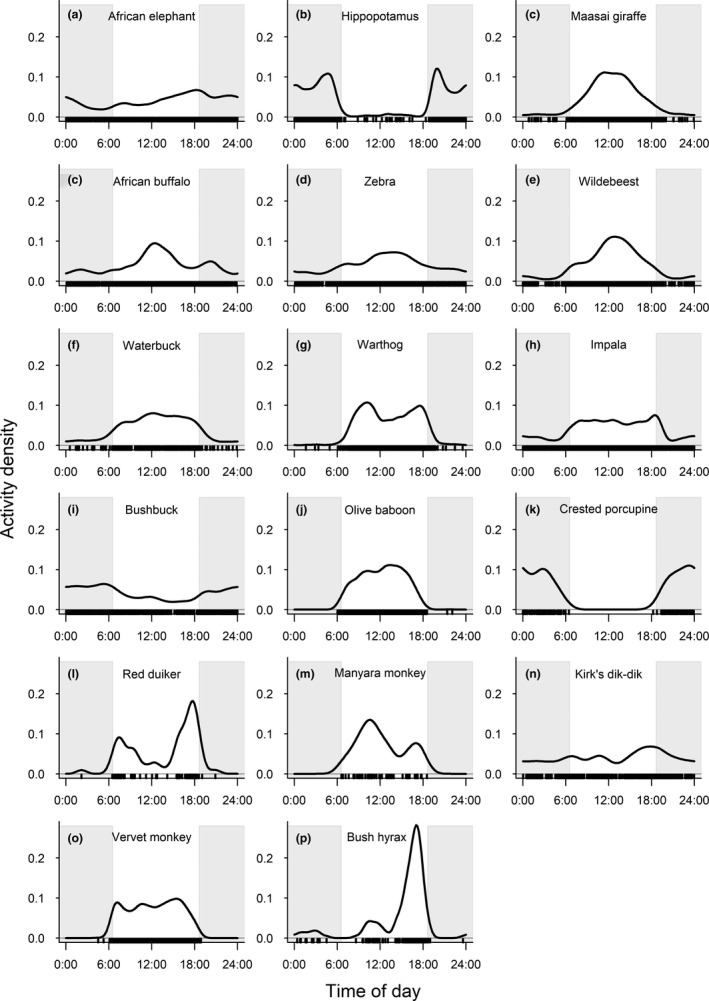
Activity patterns of herbivorous mammals in Lake Manyara National Park, Tanzania. Gray‐shaded areas represent night time, defined by the average time of sunrise and sunset during the study period

**FIGURE 5:**
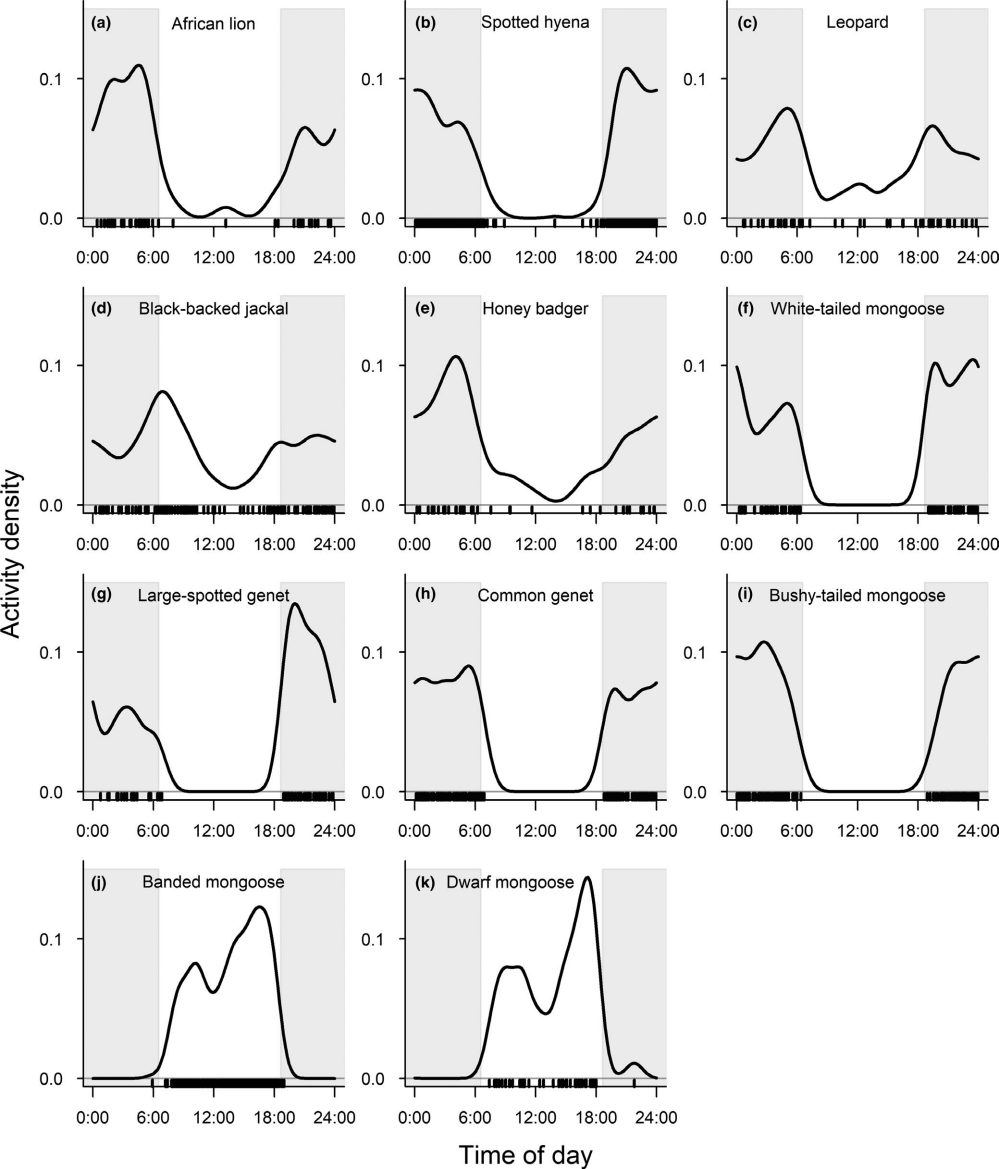
Activity patterns of faunivorous mammals in Lake Manyara National Park, Tanzania. Gray‐shaded areas represent night time, defined by the average time of sunrise and sunset during the study period

In the surveyed mammal species assemblage, both herbivore and faunivore species were either diurnal, nocturnal, or cathemeral, whereas the red duiker showed tendencies toward crepuscular behavior (Figures 4 and 5; Table 1). With the exception of hippopotamus (*Hippopotamus amphibius*), bushbuck, and crested porcupine (*Hystrix cristata*), the majority of analyzed herbivore species were most active during daytime hours (Figure 4; Table 1). In contrast, most faunivore species showed the greatest activity during nighttime hours, except for the diurnal dwarf and banded mongooses and the cathemeral black‐backed jackals (Figure 5; Table 1).

Our index of cathemerality ranged from 0.00 to 0.07 in the clearly nocturnal hippopotamus, crested porcupine, spotted hyena, large‐spotted genet, white‐tailed (*Ichneumia albicaudata*) and bushy‐tailed mongoose or the diurnal red duiker (*Cephalophus natalensis*), the three primate species, and banded and dwarf mongoose, to 0.27–0.46 in cathemeral species like elephant, buffalo (*Syncerus caffer*), impala, bushbuck, Kirk's dik‐dik (*Madoqua kirkii*), or black‐backed jackal (Table 1).

**FIGURE 6:**
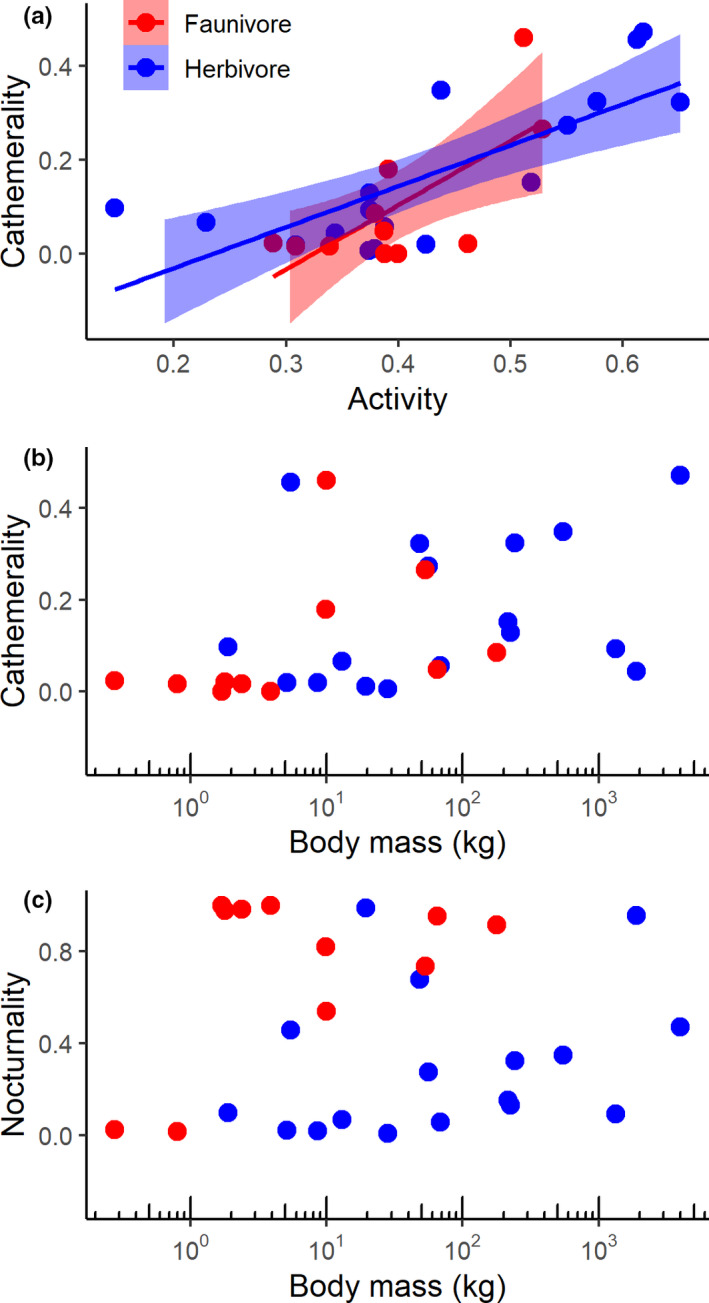
Relationship between (a) activity levels and cathemerality, (b) body mass and cathemerality, and (c) body mass and nocturnality in mammalian herbivores and faunivores of Lake Manyara National Park, Tanzania. Statistics are provided in Tables 3, 4, 5. Note that only the relationships depicted in (a) were significant

The cathemerality index increased with diel activity level (Table 3, Figure 6a), and the 95% confidence interval of the slope always included linearity. Neither trophic level nor being a ruminant had a statistically significant influence on this pattern.

**TABLE 3:** Relationship of cathemerality with diel activity level (hours active) according to y = *a* + *b* x or y = *a* + *b* x + *c*, determined by linear regression

Dataset		lambda	*a*	*p*	*b*	*p*	*c*	*p*
All	GLS	0	0.55 (0.39; 0.77)	0.002	0.97 (0.63; 1.31)	<0.001	–	–
	PGLS	0.73	0.67 (0.48; 0.95)	0.032	0.81 (0.51; 1.10)	<0.001	–	–
	GLS	0	0.58 (0.41; 0.83)	0.006	0.95 (0.61; 1.29)	<0.001	0.9 (0.8; 1.1)	0.365
	PGLS	0.74	0.67 (0.47; 0.96)	0.040	0.81 (0.50; 1.11)	<0.001	1.0 (0.7; 1.5)	0.911
Herbivores	GLS	0	0.63 (0.43; 0.92)	0.030	0.87 (0.50; 1.24)	<0.001	–	–
	PGLS	0.52	0.67 (0.46; 0.97)	0.051	0.81 (0.47; 1.15)	<0.001	–	–
	GLS	0	0.62 (0.42; 0.91)	0.029	0.82 (0.44; 1.20)	0.001	1.1 (0.9; 1.4)	0.325
	PGLS	0.47	0.66 (0.45; 0.96)	0.049	0.79 (0.44; 1.14)	0.001	1.1 (0.8; 1.6)	0.450
Faunivores	GLS	0	0.36 (0.16; 0.82)	0.037	1.38 (0.49; 2.26)	0.014	–	–
	PGLS	0.86	0.73 (0.37; 1.45)	0.397	0.74 (0.10; 1.38)	0.051	–	–

Note

For the whole dataset, *c* is the factor for trophic level (multiples of faunivore as compared to herbivore); for the herbivore dataset, *c* is the factor for digestive physiology (multiples of ruminant as compared to nonruminant). Parameters are given with their 95% confidence intervals.

### 3.3 Does cathemerality increase with body mass?

The index of cathemerality increased with body mass in the total dataset, but the scaling was no longer significant when trophic level was added to the model (Table 4, Figure 6b). Correspondingly, cathemerality did not increase with body mass within the herbivores or the faunivores. In GLS, being a ruminant was significantly associated with a higher cathemerality in the herbivores (*p* = .033), but this was no longer significant when the three primates were excluded (*p* = .180), and also not in PGLS (Table 4).

**TABLE 4:** Scaling of an index of cathemerality with body mass (BM) according to y = *a* BM*^b^* or y = *a* BM*^b^ c*, determined by linear regression on log‐transformed data

Dataset		lambda	*a*	*p*	*b*	*p*	*c*	*p*
All		0^a^	0.01 (0.00; 0.04)	<0.001	0.42 (0.12; 0.72)	0.011	–	–
		0^a^	0.02 (0.00; 0.12)	<0.001	0.35 (0.00; 0.69)	0.062	0.5 (0.1; 2.8)	0.431
Herbivores	GLS	0	0.04 (0.01; 0.14)	<0.001	0.22 (−0.07; 0.50)	0.156	–	–
	PGLS	1.00	0.08 (0.02; 0.38)	0.006	−0.02 (−0.25; 0.21)	0.845	–	–
	GLS	0	0.02 (0.01; 0.08)	<0.001	0.18 (−0.07; 0.43)	0.180	3.7 (1.3; 11.0)	0.033
	PGLS	0.90	0.06 (0.01; 0.26)	0.002	0.02 (−0.22; 0.25)	0.896	3.3 (0.4; 25.0)	0.274
Faunivores		0^a^	0.01 (0.00; 0.06)	0.001	0.61 (−0.23; 1.45)	0.188	–	–

Note

For the whole dataset, *c* is the factor for trophic level (multiples of faunivore as compared to herbivore); for the herbivore dataset, *c* is the factor for digestive physiology (multiples of ruminant as compared to nonruminant). Parameters are given with their 95% confidence intervals.

^a^ PGLS yielded the same result as GLS because lambda was estimated by maximum likelihood as zero

### 3.4 Are there positive and negative associations of nocturnality and body mass in herbivores and faunivores, respectively?

There was no scaling of nocturnality with body mass in the total dataset, unless trophic guild was included in the analysis (Table 5, Figure 6c); in the latter case, nocturnality increased with body mass and was higher in faunivores. When excluding the three primate species, the effect of body mass was no longer significant (*p* = .058 without the primates). Within the herbivores, there was no effect of body mass or being a ruminant on nocturnality. Yet, there was a trend for an increase of nocturnality with body mass in the faunivore species (Table 5). This trend was due to the two diurnal, small mongoose species (banded and dwarf mongoose) (Figure 6c).

**TABLE 5:** Scaling of nocturnality with body mass (BM) according to y = *a* BM*^b^* or y = *a* BM*^b^ c*, determined by linear regression on log‐transformed data

Dataset		lambda	*a*	*p*	*b*	*p*	*c*	*p*
All	GLS	0	0.14 (0.05; 0.38)	0.001	0.13 (−0.10; 0.37)	0.280	–	–
	PGLS	0.54	0.11 (0.03; 0.41)	0.003	0.19 (−0.06; 0.43)	0.152	–	–
		0^a^	0.04 (0.01; 0.12)	<0.001	0.33 (0.09; 0.57)	0.012	6.7 (2.1; 21.6)	0.004
Herbivores	GLS	0	0.05 (0.01; 0.20)	0.001	0.26 (−0.03; 0.56)	0.103	–	–
	PGLS	1.00	0.18 (0.03; 1.05)	0.075	0.00 (−0.26; 0.25)	0.975	–	–
	GLS	0	0.04 (0.01; 0.18)	0.001	0.25 (−0.06; 0.55)	0.131	1.7 (0.4; 6.4)	0.450
	PGLS	1.00	0.18 (0.03; 1.18)	0.095	−0.01 (−0.27; 0.26)	0.971	0.9 (0.1; 12.2)	0.920
Faunivores		0^a^	0.19 (0.07; 0.55)	0.014	0.46 (0.05; 0.87)	0.053	–	–

Note

For the whole dataset, *c* is the factor for trophic level (multiples of faunivore as compared to herbivore); for the herbivore dataset, *c* is the factor for digestive physiology (multiples of ruminant as compared to nonruminant). Parameters are given with their 95% confidence intervals in parentheses.

^a^ PGLS yielded the same result as GLS because lambda was estimated by maximum likelihood as zero.

## 4 DISCUSSION

Based on this systematic year‐long camera‐trap study, we found little support for diel activity levels to scale significantly and positively with body mass, or to differ between ruminants and nonruminants, or between herbivores and faunivores. For herbivores, the absence of a body mass scaling resembles the recent finding of Cid et al. (2020). Even for faunivores, our activity scaling exponent of 0.05 resembles that of Cid et al. (2020) for carnivores of 0.06. In contrast to their study, where the 95% CI of the exponent (0.02–0.10) was slightly above zero, zero was included in our 95% CI (−0.01 to 0.10). The low magnitude of the exponent in both studies may raise doubts about its biological relevance. Thus, in general, our findings caution against trophic level or body mass‐associated generalized conclusions regarding diel activity levels. We found that cathemerality was positively associated with diel activity levels, but—corresponding to our main finding—not consistently with body mass in the surveyed species assemblage.

### 4.1 Methodological aspects of camera trapping and activity recording

Before discussing the results, we address some methodological concerns. First, the camera‐trap placement may not capture the entire activity of species that exhibit dichotomous habitat choices such as hippopotamus, which spend the day in water, bush hyraxes which mostly live around rocks, or Manyara monkeys that are primarily arboreal. In these species, terrestrial camera‐trap placement may not represent their entire habitat niche and may thus result in biased activity patterns. In addition, our activity analyses are based on the sampled population of a species and did not differentiate between individual‐level or sex‐specific diel activity patterns, which can occur in some species such as leopards (Havmøller et al., 2020). While these considerations should be kept in mind, our community‐level analyses provide a suitable starting point to discuss general patterns of time budgets and partitioning in a large mammal assemblage. Another fundamental aspect related to the interpretation of general diel activity patterns is that they sometimes can (du Toit & Yetman, 2005) but need not necessarily parallel feeding activity; Belovsky and Slade (1986) showed that while diel activity increased with body mass in herbivores, feeding time recorded in the same individuals decreased with body mass.

### 4.2 Activity–body mass scaling

A variety of reasons can be invoked to explain why activity levels, as measured in the present study, need not increase systematically with body mass in herbivores or faunivores, and why they need not differ systematically in their overall magnitude between these trophic groups. The same considerations apply to why no systematic differences between ruminants and nonruminants were detected.

Generally, mammals of different sizes have similar energetic requirements when compared per metabolic body weight (Kleiber, 1932, 1961). Hence, if instantaneous intake capacity also scaled to metabolic body weight [for which there is indication (Shipley et al., 1994; Steuer et al., 2015)] and there were no ecological differences in the spacing and availability of food, then animals of all sizes should spend the same amount of time foraging. Note that this goes against the often‐stated rhetorical argument that it is the larger absolute energy requirements as such that necessitate more activity in larger animals (Calder, 1984; Hudson, 1985; Peters, 1983; Schmidt‐Nielsen, 1984), which has also been put forward in the context of camera‐trap investigations (Bessone et al., 2020; Cid et al., 2020).

Among species with a comparable level of metabolism, the abundance and the quality of their dietary resources should be major drivers of their foraging activity. Let us assume that animals only forage to meet their immediate requirements. Then, on the one hand, at a similar abundance of food (of similar oral processing complexity), animals specialized on higher‐quality food (such as faunivores vs. herbivores, or frugivores vs. folivores) as well as animals with a higher digestive efficiency (such as ruminant vs. nonruminant herbivores) should require less eating time and hence show less activity. On the other hand, at a similar diet quality and digestive efficiency, animals specialized in more abundant food (such as grazers vs. browsers in habitats with sparse bush and tree cover) should require less search time to locate their food. Finally, animals that acquire food in comparatively larger packages, such as large prey feeders versus invertebrate feeders among the faunivores, should have to hunt less often and therefore be able to afford to be “lazy” (De Cuyper et al., 2019; Jeschke, 2007). Thus, the interplay of food abundance, food type, and food quality can lead to various outcomes for the required foraging time, which cautions against simple body mass‐related patterns. This hypothesis corresponds to both, the absence of relevant scaling relationships in our own dataset and the equivocal results in the literature.

Yet, these considerations become even more complex if we do not follow the simplistic assumption of instantaneous requirement fulfillment, but additionally assume that animals can also use body stores to meet their requirements (Meyer et al., 2010), and that they will use surplus to invest into reproduction. Then, during times of food scarcity or low food quality, animals may either increase foraging activity to acquire the sparse resources, or decrease activity to minimize energetic losses and live off (body) stores. Similarly, during times of food abundance and higher food quality, animals may either decrease foraging activity because requirements are easily met, or they may nevertheless maintain a high foraging activity to use the ecological opportunity to build or replenish (body) stores, or to directly invest the available surplus for reproduction. There does not seem to be a straightforward way to predict how these factors will affect activity measures across animals of different body sizes, which is again reflected in the contradictory results from larger‐scale camera‐trap studies (Ramesh et al., 2015 vs. Cid et al., 2020 and the present study).

In contrast to herbivores, food search time is probably more important than ingestion time in affecting faunivore activity. Compared to herbivores, faunivores typically exhibit greater movement rates (Garland, 1983) and thus show higher activity levels during searching (Bunnell & Harestad, 1990). However, the abundance, accessibility (“catchability”), and quality of food are particularly variable in faunivores (Carbone et al., 2007). Comparative studies are further complicated by the fact that sometimes, invertebrate feeding is included in the category of “carnivory” (Carbone et al., 1999), and sometimes insectivory and carnivory are clearly distinguished (Cid et al., 2020). The relationship of diet quality to faunivore body mass depends on the predator‐prey size ratio, where it may be impossible for large insectivores to avoid some contamination of indigestible soil material (McNab, 1984), and where faunivores with a smaller or even an inverse predator–prey size ratio will not have to ingest the whole prey, but can afford to select the most nutritious parts. For example, polar bears (*Ursus maritimus*) may only consume the blubber but not the muscle meat of seals (Stirling & McEwan, 1975). By contrast, small‐prey feeders will generally ingest their prey wholly, including the less digestible fur, skin, and tendons (Rühe et al., 2008). However, the selective ingestion of prey parts in larger carnivores, and the choice of prey in general, may depend crucially on prey density and on intraguild competition (reviewed in De Cuyper et al., 2019), again making simple predictions difficult, and possibly explaining contradictory findings between different studies.

Although small and large prey‐feedings occur across the whole body size spectrum in terrestrial faunivores (De Cuyper et al., 2019), a body mass threshold of 21 kg was identified above which large prey feeding becomes more predominant (Carbone et al., 1999), because hunting of smaller prey is typically less efficient. As a result of the instantaneous surplus that faunivores can generate when hunting larger prey, models predict that they can afford to hunt less frequently and become “lazy” (De Cuyper et al., 2019; Jeschke, 2007; Rizzuto et al., 2018). The resulting activity budget distribution shows a humped shape with body mass, with a peak at a body mass of 1–10 kg (Rizzuto et al., 2018). In contrast, analyses of individual daily distance traveled (Carbone et al., 2005) and camera‐trap data (Cid et al., 2020) suggest a positive association between faunivore activity and body mass. Camera‐trap data from another study even suggest not only an increased activity in very large faunivores, but also an inversely hump‐shaped pattern, with faunivores in the 1–10 kg body mass range having the lowest daily activity (Ramesh et al., 2015). Our own data did not indicate any hump‐shaped relationship between faunivore activity levels and body mass (Figure 3). Apparently, relationships between body mass and levels of faunivore activity differ across studies.

Our results contradict a previous statement by Cid et al. (2020, p. 671) that faunivores are generally more active than herbivores based on camera‐trap data (but note that their Figure 3c indicates overlap of the 95% CI for the “intercept” estimate, similar to our estimates for *a* in our Table 2). Depending on the availability of prey in a habitat, the movement rates of faunivores—the main correlate of camera‐trap recordings (Cid et al., 2020; Rowcliffe et al., 2014)—may well be lower than those of herbivores. Supporting this interpretation, the ratio of herbivore:faunivore detections in the present study (8.6:1) was greater than the ratio of approximately 5:1 reported for global herbivore:faunivore densities (Damuth, 1987; Peters & Raelson, 1984). These observations challenge the universality of previous statements that faunivores consistently have greater movement rates than herbivores (Garland, 1983). Considering the differing predictions concerning faunivore activity and body mass, with either lower (Carbone et al., 1999; De Cuyper et al., 2019; Jeschke, 2007; Rizzuto et al., 2018) or higher (Carbone et al., 2005; Cid et al., 2020) activity at higher body masses, our results rather support the former concept. However, this should not be considered as suggesting one pattern to be more representative than the other, but rather cautioning that most likely, generalized statements on body mass‐related diel activity patterns have little predictive power.

In herbivores, diet quality has been shown to scale negatively with body mass (Clauss et al., 2013; Demment & Van Soest, 1985; Steuer et al., 2014), due to the greater abundance of fibrous plant material relative to less fibrous plant material, and that within a plant, material of different quality is spatially relatively close, so that larger body size makes the selective intake of only the nutritional parts more difficult. Because larger animals do not compensate for this lower diet quality by an increased digestive efficiency (Müller et al., 2013; Steuer et al., 2014), they have to ingest relatively more of it (Clauss et al., 2013). Whether this is achieved by higher instantaneous intake or generally longer foraging activity is difficult to predict.

Additional habitat‐specific factors might come into play. For example, du Toit and Yetman (2005) suggested that the evident discrepancy in the scaling of diel activity and foraging time with body mass between (sub)tropical and temperate ruminants could stem from differences in plant spinescence, which may force (sub)tropical browsers to generally take smaller bites and hence feed for a longer time to achieve a similar intake. Regardless of whether this explanation will stand further scrutiny, it again emphasizes that predictions based on simple allometric physiological rules are probably too simplistic. Another example on habitat specificity as explanation for variable activity levels (Mramba et al., 2019) comes from comparing our elephant activity data with those of previous studies. The LMNP elephants showed low proportional activity levels (0.54–0.68) compared to other studies that report proportional activity levels of 0.75 (Hendrichs, 1971; Wyatt & Eltringham, 1974) or even greater than 0.75 (Gravett et al., 2017). This discrepancy is likely due to abundant food resources year‐round in LMNP, facilitated by dense understory in large portions of LMNP (Kiffner et al., 2017).

Unexpectedly, we found no systematic differences in diel activity levels between ruminant and nonruminant species. Apart from measurement sensitivity, it could be that ruminants are not time‐minimizers but use the additional time to acquire resources to channel into reproduction. If we assume a higher digestive efficiency in ruminants (Clauss et al., 2015), but no reduced activity, we could speculate that bovid ruminants use that surplus to fuel their higher reproductive rates (Clauss et al., 2019; Tidière et al., 2020). Again, it is questionable that activity data from a specific habitat will allow testing of hypotheses related to general species or taxon differences related to integrative long‐term processes such as reproductive rates.

### 4.3 Diel activity patterns

Activity patterns reflect constraints imposed upon the animals by morphological, physiological, and behavioral trade‐offs, optimized vision either for day‐ or for nighttime activity (cones vs. rods), or between energy‐maximizing and time‐minimizing strategies adapted to optimize food intake versus predation risk (Owen‐Smith & Goodall, 2009; Schoener, 1971). Yet, animals can show marked changes in diel activity patterns in relation to ambient conditions, such as temperature (Nowack et al., 2020), seasonally changing food conditions (contributors to Brockman & van Schaik, 2005), and disturbance or predation pressure, possibly with long‐lasting effects ranging from individuals to whole communities (Gaynor et al., 2018; Hemingway & Bynum, 2005; Ngoprasert et al., 2017; Rasmussen, 2005). These intrinsic and extrinsic factors have often been analyzed for single species, but rarely on a community level and over a full year cycle (Zanette & Clinchy, 2020). Based on our camera‐trap study covering a multispecies assemblage in an East African national park over a complete annual cycle, we have shown that the majority of mammal species (21/28 if considering a 20% threshold for cathemerality; 23/28 if considering a 30% threshold for cathemerality) exhibited circadian activity patterns that are consistent with published patterns for these species (Table 1). However, diel activity pattern categories of 18% (30% threshold for cathemerality) to 25% (20% threshold for cathemerality) of species differed from patterns depicted in a global database of mammalian activity patterns (Bennie et al., 2014), questioning the generality of conclusions drawn from large‐scale analyses and highlighting the need to carry out fieldwork to generate location‐based insights on species‐specific circadian rhythms. The majority of discrepancies (5/7 considering the 20% threshold for cathemerality) were species classified as nocturnal in the global database, but actually exhibited cathemeral or diurnal activity patterns in LMNP (Table 1).

Generally, it is assumed that larger animals are more likely to be cathemeral, due to a presumed increase of activity levels with body size (van Schaik & Griffiths, 1996). Under the assumption that herbivores are generally diurnal and faunivores generally nocturnal (Wu et al., 2018), this translated into an expectation that larger species should have a lesser proportion of their preferred diel activity window; in other words, we expected herbivores to become more nocturnal with body mass, and faunivores less so. For the herbivores, this expectation was not met (Table 5): the relatively small‐sized bushbucks, the porcupines, and dik‐diks had high proportions of nocturnal activity. For the faunivores, this expectation was also not met, but the result was actually the opposite, due to the two smallest mongoose species being nearly completely diurnal. Yet again, these findings emphasize a discrepancy between global patterns and those detectable at the level of a specific community, indicating that community‐specific data rather than extrapolations from the former should guide understanding of a specific animal community.

As expected, cathemerality was clearly associated with the level of activity in our dataset—but, similar to activity itself, not with body mass. Again, our findings do not concur with simple body mass‐related assumptions. For cathemerality, it should not be forgotten that some very small animals, which require an ultradian activity rhythm because they cannot sustain a 12‐hr fast, are also expected to be cathemeral (van Schaik & Griffiths, 1996).

Several diurnal species (especially those with lower activity levels) may not have such adaptive potential along the time axis, yet face the dual challenge to adjust their activity patterns to human activities and those of competitors and predators (Frey et al., 2020; Haswell et al., 2020; Shamoon et al., 2018). In LMNP, where human disturbance is limited to photographic tourism (which mostly occurs during daytime) and occasional illegal hunting (which mostly occurs during night time), the majority of herbivores was primarily diurnal whereas the majority of faunivores was primarily nocturnal (Table 1). Mammals tend to be more nocturnal if subject to greater intensities of the human footprint (Gaynor et al., 2018) and the already substantial proportion of nocturnal activity in carnivores (especially large bodied species) may make the faunivore community in the wider Tarangire–Manyara ecosystem relatively less sensitive to human interference by different land use compared to the herbivore community (Msuha et al., 2012).

## 5 CONCLUSIONS

Our results do not support the conclusions of previous studies on diel activity patterns in tropical mammals in relation to body mass (Cid et al., 2020; Owen‐Smith & Goodall, 2009; Ramesh et al., 2015), and thus caution against trophic level or body mass‐associated generalized conclusions with regard to activity patterns. As camera‐trap data typically model activity patterns at the population level (Cid et al., 2020; Rowcliffe et al., 2014) and thus ignore individual or sex‐related differences in behavior (Havmøller et al., 2020), and may not cover the entire habitat niche of several species activity pattern, analyses based on camera‐trap data should best be combined with behavioral data sampled at the resolution of individual animals in order to arrive at more comprehensive results.

## CONFLICT OF INTEREST

None declared.

## AUTHOR CONTRIBUTIONS

**Marcus Clauss:** Conceptualization (lead); formal analysis (equal); investigation (lead); methodology (lead); software (equal); visualization (lead); writing–original draft (lead). **Miriam Scriba:** Data curation (equal); formal analysis (equal); investigation (equal); methodology (equal); software (equal); visualization (equal); writing–review and editing (supporting). **John Kioko:** Data curation (supporting); funding acquisition (lead); investigation (equal); project administration (equal); supervision (equal); validation (equal); writing–review and editing (equal). **Jörg U. Ganzhorn:** Conceptualization (supporting); formal analysis (supporting); supervision (supporting); validation (lead); writing–original draft (supporting); writing–review and editing (lead). **Christian Kiffner:** Conceptualization (lead); data curation (lead); formal analysis (lead); funding acquisition (equal); investigation (lead); methodology (equal); project administration (lead); software (equal); supervision (lead); visualization (lead); writing–original draft (equal).

## Data Availability

Data to reproduce the main analyses of this paper are presented in Table 1. The raw data (time‐stamped independent detection of all considered species) are available at: https://doi.org/10.25625/T7A9NW.
